# Effects of Oral Xylitol, Sucrose, and Acesulfame Potassium on Total Energy Intake During a Subsequent *ad libitum* Test Meal: A Randomized, Controlled, Crossover Trial in Healthy Humans

**DOI:** 10.3390/nu17030484

**Published:** 2025-01-29

**Authors:** Emilie Flad, Anita Altstädt, Christoph Beglinger, Jens F. Rehfeld, Lukas Van Oudenhove, Bettina K. Wölnerhanssen, Anne Christin Meyer-Gerspach

**Affiliations:** 1 St. Clara Research Ltd. at St. Claraspital, 4002 Basel, Switzerland; 2 Faculty of Medicine, University of Basel, 4001 Basel, Switzerland; 3 Department of Clinical Biochemistry, Rigshospitalet, University of Copenhagen, 1172 Copenhagen, Denmark; 4 Laboratory for Brain-Gut Axis Studies, Translational Research Center for Gastrointestinal Disorders, Department of Chronic Diseases and Metabolism, KU Leuven, 3000 Leuven, Belgium; 5 Leuven Brain Institute, KU Leuven, 3000 Leuven, Belgium; 6 Cognitive and Affective Neuroscience Laboratory, Department of Psychological and Brain Sciences, Dartmouth College, Hanover, NH 03755, USA

**Keywords:** xylitol, sucrose, acesulfame potassium, energy intake, gastrointestinal satiation hormones, appetite-related sensations, healthy participants

## Abstract

Background/Objectives: Xylitol, a natural low-caloric bulk sweetener, is increasingly used as a sugar alternative due to its low-glycemic and low-insulinemic properties. The aim was to investigate the effect of orally administered xylitol, sucrose, and acesulfame potassium (ace-K) on energy intake during a subsequent *ad libitum* test meal. Methods: In this randomized, controlled, double-blind, crossover trial (ClinicalTrials.gov NCT05671965, 20 December 2022), we included 20 healthy participants with normal body weight. Over four study visits, participants consumed an oral preload containing 33.5 g xylitol, 33.5 g sucrose, or 0.1675 g ace-K dissolved in 300 mL water, or 300 mL pure water as control. Participants were provided with an *ad libitum* test meal 15 min after the preload consumption, and both energy intake and total energy intake (= preload + *ad libitum* test meal) were assessed. Blood samples were collected to quantify cholecystokinin (CCK), glucagon-like peptide-1 (GLP-1), glucose, and insulin concentrations. Results: Total energy intake was lower in response to xylitol and ace-K compared to sucrose (*p_Tukey_* < 0.03), with no differences between xylitol and ace-K or water. Plasma CCK concentrations were higher in response to xylitol compared to sucrose, ace-K, and water (*p_Holm_* < 0.01), whereas GLP-1 concentrations did not differ between the preloads. Plasma glucose and insulin concentrations were lower in response to xylitol compared to sucrose (*p_Holm_* < 0.01), but xylitol led to an increase in insulin compared to ace-K and water (*p_Holm_* < 0.01). Conclusions: The consumption of oral preloads sweetened with xylitol or ace-K prior to an *ad libitum* test meal result in a lower total energy intake compared to a preload with sucrose.

## 1. Introduction

The rising prevalence of overweight and obesity has become a critical public health issue in the 21st century. One of the key contributors to this epidemic is the excessive daily intake of sugar, particularly through sugar-sweetened beverages (SSBs) [1]. Recently, the European Food Safety Authority (EFSA) declared that, based on current evidence, no safe level of sugar intake can be determined, recommending that sugar consumption be kept as low as possible [2].

The most popular sugar is sucrose, known as table sugar. Sucrose has satiating properties by stimulating the release of the gastrointestinal (GI) satiation hormones glucagon-like peptide-1 (GLP-1) and glucose-dependent insulinotropic polypeptide (GIP), while also slowing gastric emptying [3,4,5,6]. Numerous studies have examined its satiating effects on energy intake during subsequent test meals, yielding mixed results. Some studies observed no differences in energy intake but noted a higher total energy intake compared to artificial low-caloric high-intensity sweeteners (LC-HIS) or water, attributable to sucrose’s caloric content (4 kcal/g) [7,8]. Conversely, other studies reported a lower energy intake following sucrose consumption compared to other sweeteners, along with a similar or lower total energy intake, respectively [5,9,10,11]. The results from chronic intervention studies are also inconsistent. A 10-week supplementation with sucrose-containing drinks and foods resulted in a higher total energy intake and body weight, whereas in another study, six months of daily sucrose consumption did not affect total energy intake [12,13].

Artificial low-caloric high-intensity sweeteners, such as acesulfame potassium (ace-K), aspartame, and sucralose, have gained popularity as sugar alternatives. However, artificial LC-HIS do not induce satiety, since they neither stimulate the release of GI satiation hormones nor delay gastric emptying [14,15,16]. Research investigating the effect of artificial LC-HIS on energy intake has produced mixed results, with studies reporting decreased, unchanged, or even increased energy intake [17,18]. Additionally, the effect of chronic artificial LC-HIS consumption on glucose homeostasis remains unclear, with some interventional and observational studies suggesting that these sweeteners may be associated with an increased risk of developing type 2 diabetes mellitus (T2DM) [19,20,21,22,23]. It is important to note that while observational studies may identify potential relationships between artificial LC-HIS and metabolic diseases, they do not establish causality. Therefore, future chronic interventional studies are necessary.

Natural low-caloric bulk sweeteners such as the five-carbon sugar alcohol erythritol and the four-carbon sugar alcohol xylitol present an alternative to artificial LC-HIS and sugars. While both xylitol and erythritol belong to the class of sugar alcohols, their physicochemical properties differ significantly, which may influence their metabolic effects and applications. For instance, erythritol is almost completely absorbed in the small intestine and excreted unchanged via urine, whereas xylitol undergoes partial fermentation in the colon. Xylitol has a sweetness comparable to sucrose but with only 60% of the calories (2.4 kcal/g) [24]. Beyond their low-glycemic and low-insulinemic effects, xylitol and erythritol are known to induce the release of GI satiation hormones—including cholecystokinin (CCK), GLP-1, and peptide tyrosine tyrosine (PYY)—and delay gastric emptying rates (for a review, see [19]). Based on these satiating properties, we previously investigated the influence of erythritol on energy intake during a subsequent *ad libitum* test meal, in comparison to sucrose, sucralose, and tap water [25]. Our findings demonstrated that in response to erythritol, total energy intake (preload + *ad libitum* test meal) and even the energy intake during the subsequent *ad libitum* test meal were reduced. To date, only two human studies have investigated the effect of xylitol on energy intake: Shafer et al. [26] measured energy intake during an *ad libitum* buffet in response to preloads containing xylitol, sucrose, glucose, fructose, or water, and found a lower energy intake in response to xylitol compared to water and no difference in energy intake in response to sucrose, glucose, and fructose compared to water. King et al. [27] investigated the effect of the daily consumption of yoghurts sweetened with xylitol, polydextrose, xylitol + polydextrose, or sucrose over ten days on energy intake during an *ad libitum* lunch. They found no significant difference in energy intake in response to xylitol compared to sucrose; however, considering the caloric content of the yoghurts, a lower total energy intake was observed [27].

The primary aim of this study was to explore the effect of xylitol, sucrose, and ace-K consumption on energy intake during a subsequent *ad libitum* test meal as well as on total energy intake. Secondary aims included evaluating the effects of xylitol on the release of GI satiation hormones (CCK, GLP-1), parameters of glycemic control (glucose, insulin), appetite-related sensations, and GI tolerance.

Based on previous research, we hypothesized that xylitol and sucrose will lead to a lower energy intake during the *ad libitum* test meal compared to ace-K and water.

## 2. Materials and Methods

### 2.1. Ethical Statement

The regional ethics board (Ethikkommission Nordwest- und Zentralschweiz (EKNZ)) approved the study on 3 January 2023 (BASEC-Nr. 2022-02165). The trial was conducted according to the guidelines of the current version of the Declaration of Helsinki (DoH-Oct2024) and national legal and regulatory requirements. All participants provided written informed consent before participating in the study. The study was registered at ClinicalTrials.gov (NCT05671965) on 20 December 2022.

### 2.2. Participants

Twenty participants (10 women, 10 men) completed the study. Eligibility for participation required meeting the following inclusion criteria: age between 18 and 55 years, body mass index (BMI) between 19.0 and 24.9 kg/m^2^, and stable body weight (±5%) for at least three months prior to the study. Exclusion criteria included fructose intolerance, adherence to specific diets (such as vegetarian, vegan, sugar free, and/or no breakfast), the regular consumption of xylitol and/or ace-K (>1/week), the presence of chronic or clinically relevant acute infections or diseases, regular medication use, pregnancy, alcohol or substance abuse, working night shifts, and participation in another clinical study within the 30 days preceding or during the present study.

### 2.3. Study Design and Procedure

**Figure 1:**
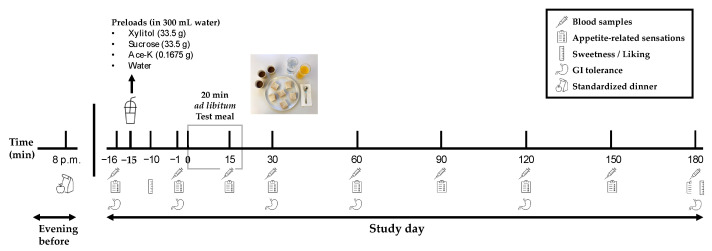
Study design. Participants consumed a standardized dinner by 8 p.m. the evening before the study visit and fasted overnight. After the collection of a fasting blood sample and baseline ratings of appetite-related sensations and GI symptoms, participants received one of four oral preloads in a randomized order: 33.5 g xylitol, 33.5 g sucrose, or 0.1675 g ace-K dissolved in 300 mL water, or 300 mL pure water. Ratings of sweetness and liking of the preloads were collected. Fifteen minutes later (t = 0 min), the *ad libitum* test meal was served for 20 min. At fixed timepoints, blood samples and ratings of appetite-related sensations and GI symptoms were collected. At t = 180 min, rating of liking of the test meal was collected. Ace-K, acesulfame potassium; GI, gastrointestinal.

This study was a randomized, controlled, double-blind, crossover study, comprising one screening visit and four study visits (Figure 1). The study design was adapted from our previous study investigating the effects of oral erythritol on energy intake [25]. All study visits took place at St. Clara Research Ltd., Basel, Switzerland, between February and June 2023. In the 24 h prior to each study visit, participants were instructed to avoid physical activities, alcohol, and caffeine, and to consume a standardized dinner (consisting of a soup, spaghetti bolognese, and a chocolate bar) by 8 p.m. Participants then underwent a 12 h overnight fast from 8 p.m. until the start of the study visit at 8 a.m.

At the beginning of the study visit, a catheter was inserted into a forearm vein, and fasting blood samples (t = −16 min) were collected. Participants then received one of the following oral preloads (t = −15 min), which they were required to consume within two minutes:

33.5 g xylitol dissolved in 300 mL water;

33.5 g sucrose dissolved in 300 mL water;

0.1675 g ace-K dissolved in 300 mL water;

300 mL water (control).

Fifteen minutes after the preload (t = 0 min), participants were served a standard test meal and were instructed to eat and drink *ad libitum*. The test meal ended after 20 min or when the participant had stopped eating and/or drinking for more than five minutes. To minimize awareness of the amount eaten, the food and drinks were refilled irregularly and in excess. Further blood samples were collected before the start of the *ad libitum* test meal (t = −1 min) as well as at regular time points during and after the test meal (t = 15, 30, 60, 90, 120, 150, and 180 min) to measure GI satiation hormones (CCK and GLP-1_total_) and parameters of glycemic control (glucose and insulin). Furthermore, appetite-related sensations (hunger, prospective food consumption (pfc), satiety and fullness) were assessed at t = −16, −1, 15, 30, 60, 90, 120, 150, and 180 min using visual analogue scales (VASs) [28,29]. GI tolerance was assessed at t = −16, −1, 30, 60, 120, and 180 min using a symptom checklist (0, no symptoms; 1, mild symptoms; 2, severe symptoms) including the following symptoms: abdominal pain, nausea, vomiting, diarrhea, borborygmus, abdominal distension, eructation, and flatulence. Perceived sweetness and liking of the preloads (t = −10 min) and the test meal (t = 180 min) were measured using Global Sensory Intensity Scale (GSIS) and Global Hedonic Intensity Scale (GHIS), respectively [30].

### 2.4. Composition and Conduction of the Preload and Test Meal

#### 2.4.1. Preload

The oral preloads were freshly prepared every morning prior to the study visits and served at room temperature. The doses of xylitol (33.5 g), sucrose (33.5 g), and ace-K (0.1675 g) were matched to be equisweet, comparable to a standard sweetened beverage of around 300 mL. To ensure the blinding of both the study personnel and the participants, the preloads were prepared by a colleague not involved in the study’s execution or data analysis. The order in which the preloads were administered was randomized and counterbalanced across the four study visits. The sweeteners were purchased from the following suppliers:

Xylitol: Schweizer Edelzucker AG, St. Gallen, Switzerland;

Sucrose: Hänseler AG, Herisau, Switzerland;

Ace-K: Sigma-Aldrich, Buchs, Switzerland.

#### 2.4.2. *Ad libitum* Test Meal

The *ad libitum* test meal was freshly prepared every morning prior to the study visit by the study personnel. The drink options included a glass of water (250 mL, 0 kcal/100 mL) and a glass of cooled orange juice (250 mL, 40 kcal/100 mL). The food options consisted of cups of chocolate cream (50 g, 128 kcal/100 g) and ham sandwich squares. The ham sandwiches were made with two slices of toast (56 g, 265 kcal/100 g), butter (10 g, 742 kcal/100 g), and one slice ham (12.5 g, 97 kcal/100 g). Each sandwich was cut into four equal squares, with each square weighing 19.6 g and containing 59 kcal. The test meal was individually served and, throughout the test meal, food and drinks were replenished irregularly and in excess. This approach ensured that food and drinks never depleted and minimized the participant’s awareness of how much they consumed.

All food items were purchased in a local supermarket, and orange juice, chocolate cream, butter, and ham were stored in the fridge at 7 °C.

### 2.5. Assessment of Energy Intake and Total Energy Intake

Energy intake during the *ad libitum* test meal was calculated by measuring the difference in the number of sandwich squares and cups of chocolate cream, as well as the difference in the volume of water and orange juice before and after the test meal. Total energy intake was determined by considering the calories from both the preload (xylitol preload: 80 kcal, sucrose preload: 134 kcal) and the *ad libitum* test meal.

### 2.6. Blood Sample Collection and Processing

For the analysis of CCK and GLP-1_total_, blood was collected on ice into tubes containing ethylenediaminetetraacetic acid (EDTA) (6 µmol/L blood), a protease-inhibitor cocktail (Complete, EDTA-free, 1 tablet/50 mL blood, Roche, Mannheim, Germany), and a dipeptidyl peptidase IV (DPPIV) inhibitor (10 µL/mL blood, Millipore Corp., St. Charles, MO, USA).

For the analysis of *glucose and insulin*, blood was also collected on ice into tubes containing EDTA (6 µmol/L blood) and the protease-inhibitor cocktail Complete (EDTA-free, 1 tablet/50 mL blood).

All blood samples were centrifuged (4 °C, 3000 rpm, 10 min), and the plasma was aliquoted and stored at −80 °C until analysis.

### 2.7. Laboratory Analyses

Plasma CCK concentration was measured with a sensitive radioimmunoassay using a highly specific antiserum (No. 92128) [31]. The intra and inter-assay variability is below 15% and the appropriate range of the assay is 0.1 to 20 pmol/L. Plasma GLP-1_total_ concentration was measured with a non-radioactive, highly sensitive sandwich ELISA (MSD V-PLEX-#K1503PD) in the presence of a chemiluminescent substrate. The intra- and inter-assay variability is 0.18 to 120 pM when using a 50 µL sample size. Plasma glucose concentration was measured with an enzymatic assay from Beckman Coulter (Rothen Medizinische Laboratorien AG, Basel, Switzerland). The intra- and inter-assay variability is below 0.8% and 1.4% and the appropriate range is 0.11 to 41.6 mmol/L. Plasma insulin concentration was measured with an electrochemiluminescence immunoassay (ECLIA) (Rothen Medizinische Laboratorien AG, Basel, Switzerland). The intra- and inter-assay variability is below 1.5% and 4.9% and the appropriate range is 0.2 to 1000 µIU/mL.

### 2.8. Statistical Analysis

A previous study using lower doses of xylitol detected a large effect size (d = 1.01) for the difference in energy intake [26]. This study had only a small sample size (*n* = 9), which is why the sample size in our study was conservatively calculated based on a medium effect size (f = 0.34), with *n* = 20 yielding 95% power to detect a similar difference in the omnibus test of the mixed ANOVA, comparing *ad libitum* energy intake after the four preloads.

The statistical data analysis was conducted in SAS 9.4 (SAS Institute, Cary, NC, USA). Data are presented as mean ± standard deviation (SD) unless otherwise specified. Statistical significance was defined as *p* < 0.05, with values between 0.05 and 0.1 considered as trends. The normality of the data was assessed using the Shapiro–Wilk test, and variables were Box–Cox transformed when necessary to achieve a normal distribution. Linear mixed models were used to analyze the outcome variables, using either absolute values (e.g., energy intake, total energy intake, sweetness, and liking) or changes from baseline (e.g., GI satiation hormones, glycemic control, and appetite-related sensations). Homoscedasticity was assessed by inspecting the residuals of the respective linear mixed models. The visit number was included to account for potential order effects. “Preload” and “time” (for GI satiation hormones, glycemic control, and appetite-related sensations) were considered as within-subject independent variables in the models including their main effects and the interaction. Additionally, all models for GI satiation hormones, glycemic control, and appetite-related sensations were controlled for “total energy intake”. Planned contrast analyses were performed to test our specific hypothesis using Student’s *t*-tests with Tukey correction (for energy intake, total energy intake, sweetness, and liking) or stepdown Bonferroni–Holm correction (for GI satiation hormones, glycemic control, and appetite-related sensations) to correct for multiple testing:

- A comparison of the energy intake between xylitol, sucrose, ace-K, and water to test the hypothesis that xylitol and sucrose will lead to a lower energy intake during the *ad libitum* test meal compared to ace-K and water.
- A comparison of the total energy intake between xylitol, sucrose, ace-K, and water to test the hypothesis that xylitol will lead to a lower total energy intake compared to sucrose, ace-K, and water.
- A comparison of post-preload administration time point t = −1 min versus baseline values for each preload to test the hypotheses that: (1) CCK and GLP-1_total_ will be released in response to xylitol and sucrose, but not in response to ace-K or water; (2) glucose and insulin concentrations will be increased in response to sucrose, but not in response to xylitol, ace-K, or water; and (3) hunger/pfc will be decreased and satiety/fullness will be increased in response to xylitol and sucrose, but not in response to ace-K or water.
- A comparison of post-preload administration time point t = −1 min versus baseline between xylitol and sucrose, ace-K, or water to test the hypotheses that: (1) CCK and GLP-1_total_ in response to xylitol will be similar to sucrose, but higher compared to ace-K or water; (2) glucose and insulin concentrations will be lower in response to xylitol compared to sucrose, but similar to ace-K or water; (3) hunger/pfc and satiety/fullness, respectively, in response to xylitol will be similar to sucrose, but lower and higher, respectively, compared to ace-K or water.
- A comparison of post-preload administration time point t = 15 min (during the *ad libitum* test meal) versus baseline values between xylitol, sucrose, ace-K, or water to explore GI satiation hormones, glycemic control, and appetite-related sensations. No hypotheses were formulated beforehand.
- A comparison of perceived sweetness, the liking of preloads, and liking of the test meal between xylitol and sucrose, ace-K, or water to test the hypothesis that xylitol will have a similar perceived sweetness as sucrose and ace-K, but it will be higher compared to water. No differences will be observed in the liking of preloads and the test meal in response to xylitol compared to sucrose, ace-K, or water.

Spearman’s correlation was used to explore the relationship between differences in the post-preload administration time point (t = −1 min) plasma CCK concentrations for xylitol and ace-K, and the corresponding difference in energy intake.

## 3. Results

**Figure 2:**
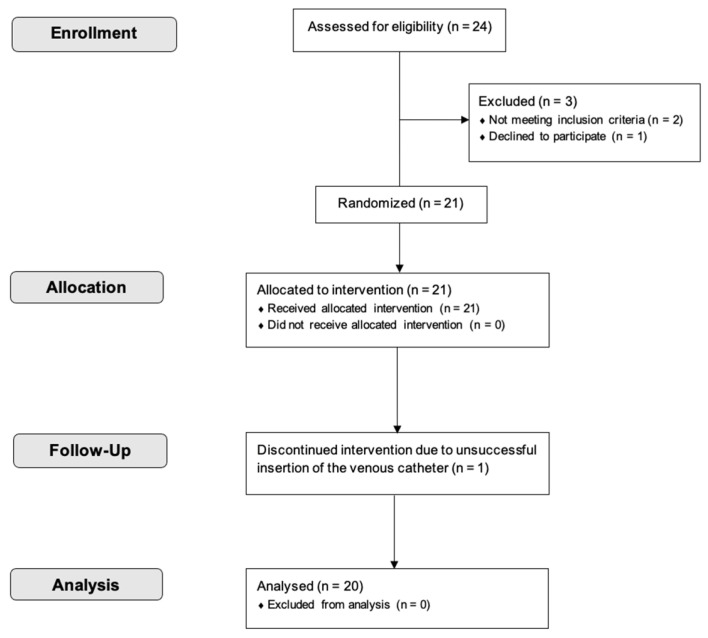
CONSORT flow diagram.

Twenty-one participants were initially included in this study. However, one participant dropped out due to unsuccessful venous catheter insertion on two separate days. As a result, complete data sets were obtained from 20 participants (10 women, 10 men; mean ± SD (range); age: 27.5 ± 7.8 (21–54) years; BMI: 23.0 ± 1.4 (19.9–24.7) kg/m^2^) and used for the final analysis (Figure 2).

### 3.1. Energy Intake and Total Energy Intake

**Figure 3:**
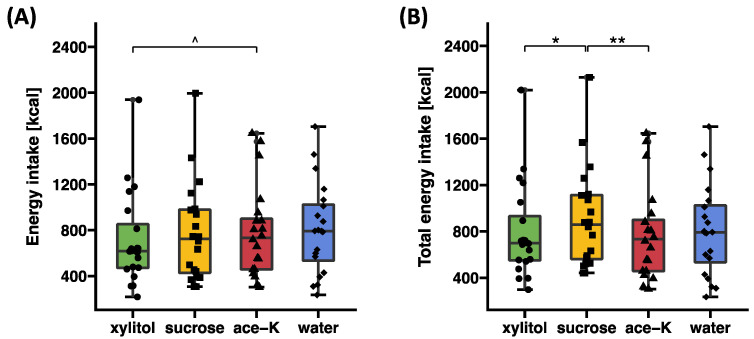
(**A**) Energy intake (*ad libitum* test meal) and (**B**) total energy intake (preload + *ad libitum* test meal) in response to oral preloads containing 33.5 g xylitol, 33.5 g sucrose, 0.1675 g ace-K, or water. Data are expressed as median and interquartile range with individual values. Statistical tests: linear mixed models followed by planned contrast analysis with Tukey correction for multiple testing. Ace-K, acesulfame potassium. Significance: ^ *p_Tukey_* < 0.1; * *p_Tukey_* < 0.05; ** *p_Tukey_* < 0.01. N = 20.

Figure 3 and Table 1 present energy intake (*ad libitum* test meal) and total energy intake (preload + *ad libitum* test meal) in response to preloads containing xylitol, sucrose, ace-K, or water.

The main effect of preload was significant on both energy intake (F(3,19) = 4.28, *p* =0.018) and total energy intake (F(3,19) = 7.09, *p* = 0.002). Planned contrast analyses revealed that energy intake tended to be lower in response to xylitol compared to ace-K (*p_Tukey_* = 0.076), with no significant differences between xylitol and sucrose (*p_Tukey_* = 0.522), xylitol and water (*p_Tukey_* = 0.171), sucrose and ace-K (*p_Tukey_* = 0.899), sucrose and water (*p_Tukey_* = 0.847), and ace-K and water (*p_Tukey_* = 0.989). Additionally, planned contrast analyses revealed that total energy intake was significantly lower in response to xylitol (*p_Tukey_* = 0.029) and ace-K (*p_Tukey_* = 0.003) compared to sucrose, with no significant differences between xylitol and ace-K (*p_Tukey_* = 0.930), xylitol and water (*p_Tukey_* = 1.000), sucrose and water (*p_Tukey_* = 0.132), and ace-K and water (*p_Tukey_* = 0.989).

### 3.2. GI Satiation Hormones: Plasma CCK and GLP-1_total_

**Figure 4:**
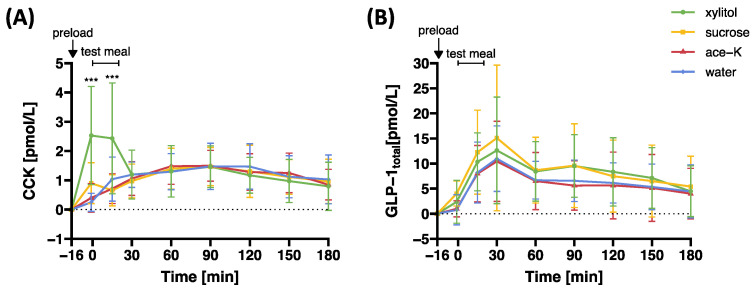
Plasma (**A**) CCK and (**B**) GLP-1_total_ concentrations in response to oral preloads containing 33.5 g xylitol, 33.5 g sucrose, 0.1675 g ace-K, or water. Data are expressed as mean ± SD and differences to baseline values are reported. Statistical tests: linear mixed models followed by planned contrast analysis with Holm correction for multiple testing. Ace-K, acesulfame potassium; CCK, cholecystokinin; GLP-1, glucagon-like peptide-1. Significance: *** *p_Holm_* < 0.001. N = 20.

Figure 4 and Table 2 present plasma concentrations of CCK and GLP-1_total_ in response to preloads containing xylitol, sucrose, ace-K, or water.

The main effect of preload on CCK was significant (F(3,63.7) = 3.95, *p* = 0.012), as was the preload-by-time interaction effect (F(21,287) = 7.69, *p* < 0.001). Planned contrast analyses revealed that plasma CCK concentrations were significantly higher in response to xylitol compared to sucrose, ace-K, or water both before (t = −1 min) and during (t = 15 min) the *ad libitum* test meal (*p_Holm_* < 0.001 for all comparisons).

Neither the main effect of preload on GLP-1_total_ (F(3,61.6) = 2.00, *p* = 0.123) nor the preload-by-time interaction effect (F(21,284) = 0.71, *p* = 0.825) were significant. None of the planned contrast analyses for GLP-1_total_ were significant.

### 3.3. Associations Between CCK Release and Energy Intake

No significant associations were found between the differences in plasma CCK concentrations and the differences in energy intake in response to xylitol and ace-K (*r_s_* = 0.087; *p* = 0.714).

### 3.4. Glycemic Control: Plasma Glucose and Insulin

**Figure 5:**
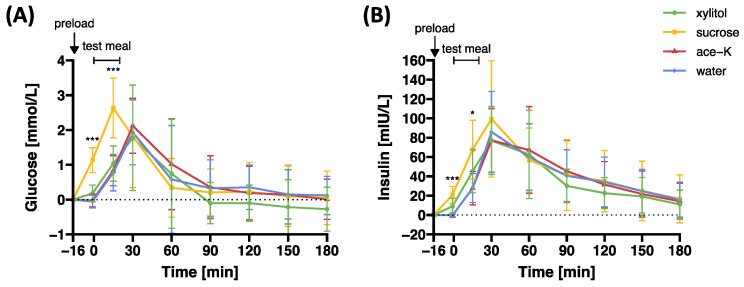
Plasma (**A**) glucose and (**B**) insulin concentrations in response to oral preloads containing 33.5 g xylitol, 33.5 g sucrose, 0.1675 g ace-K, or water. Data are expressed as mean ± SD and differences to baseline values are reported. Statistical tests: linear mixed models followed by planned contrast analysis with Holm correction for multiple testing. Ace-K, acesulfame potassium. Significance: * *p_Holm_* < 0.05; *** *p_Holm_* < 0.001. N = 20.

Figure 5 and Table 2 present plasma concentrations of glucose and insulin in response to preloads containing xylitol, sucrose, ace-K, or water.

The main effect of preload on glucose was significant (F(3,69.8) = 4.38, *p* = 0.007), as was the preload-by-time interaction effect (F(21,295) = 5.04, *p* < 0.001). Planned contrast analyses revealed that plasma glucose concentrations were significantly lower in response to xylitol compared to sucrose both before (t = −1 min) and during (t = 15 min) the *ad libitum* test meal (*p_Holm_* < 0.001 for both comparisons), with no significant differences between xylitol and ace-K or water (*p_Holm_* > 0.367 for all comparisons).

The main effect of preload on insulin was significant (F(3,43.5) = 6.05, *p* = 0.002), as was the preload-by-time interaction effect (F(21,253) = 13.17, *p* < 0.001). Planned contrast analyses revealed that before the test meal (t = −1 min), plasma insulin concentrations were significantly lower in response to xylitol compared to sucrose but significantly higher in response to xylitol compared to ace-K or water (*p_Holm_* < 0.001 for all comparisons). During the *ad libitum* test meal (t = 15 min), there were no significant differences in plasma insulin concentrations between xylitol and sucrose or water (*p_Holm_* > 0.059 for both comparisons). However, plasma insulin concentrations were significantly higher in response to xylitol compared to ace-K (*p_Holm_* = 0.032).

### 3.5. Appetite-Related Sensations: Hunger, Prospective Food Consumption (Pfc), Satiety, and Fullness

Table 2 presents appetite-related sensations in response to the preloads containing xylitol, sucrose, ace-K, or water.

Neither the main effects of preload on hunger, pfc, satiety and fullness were significant (F(3,61.6) = 1.20, *p* = 0.317, F(3,62.2) = 1.93, *p* = 0.135, F(3,61.8) = 0.66, *p* = 0.581, and F(3,59.4) = 1.62, *p* = 0.195, respectively) nor the preload-by-time interaction effects (F(21,286) = 0.78, *p* = 0.744, F(21,288) = 0.65, *p* = 0.878, F(21,290) = 0.85, *p* = 0.659, and F(21,284) = 0.68, *p* = 0.850, respectively). Additionally, none of the planned contrast analyses revealed significant results.

### 3.6. Sweetness and Liking Ratings: Preload and Test Meal

The main effect of preload on ratings of perceived sweetness of the preloads was significant (F(3,19) = 167.50, *p* < 0.001). Planned contrast analyses revealed no significant differences in ratings of perceived sweetness between xylitol and sucrose (*p_Tukey_* = 0.377); however, the rating of perceived sweetness of xylitol was significantly higher compared to ace-K (*p_Tukey_* = 0.020) or water (*p_Tukey_* < 0.001).

There was a trend in the main effect of preload on ratings of liking for the preloads (F(3,19) = 2.47, *p* = 0.093).

The main effect of preload on liking ratings for the test meal was significant (F(3,19) = 6.18, *p* = 0.004). Planned contrast analyses revealed no significant difference in the liking ratings between xylitol and sucrose (*p_Tukey_* = 0.659); however, the liking for the test meal tended to be lower in response to xylitol compared to ace-K (*p_Tukey_* = 0.073) and was significantly lower compared to water (*p_Tukey_* = 0.013).

### 3.7. GI Tolerance

Table 3 presents the GI symptoms in response to the preloads containing xylitol, sucrose, ace-K, or water.

Seven participants reported mild borborygmi, and three participants experienced diarrhea in response to xylitol; however, all symptoms resolved during or shortly after the study visit. Overall, participants tolerated the study well, with no withdrawals due to GI symptoms.

## 4. Discussion

In this randomized, controlled, double-blind, crossover trial, we investigated the effects of oral preloads containing xylitol, sucrose, ace-K, or water on energy intake and total energy intake during a subsequent *ad libitum* test meal. Furthermore, plasma concentrations of the GI satiation hormones CCK and GLP-1_total_, as well as plasma glucose and insulin concentrations, were measured. Participants’ appetite-related sensations, as well as their ratings of perceived sweetness and liking for preloads and the *ad libitum* test meal, were also recorded. The results show that (1) energy intake during the *ad libitum* test meal was similar in response to all preloads; (2) total energy intake (preload + *ad libitum* test meal) was significantly lower in response to xylitol and ace-K compared to sucrose, with no significant differences to water; (3) plasma CCK concentrations were significantly higher in response to xylitol compared to sucrose, ace-K, and water; and (4) plasma glucose concentrations were significantly lower in response to xylitol compared to sucrose with no differences to ace-K or water, whereas insulin concentrations were significantly lower in response to xylitol compared to sucrose but significantly higher compared to ace-K or water.

The natural low-caloric bulk sweetener xylitol has become popular as an alternative sweetener, due to its low caloric content, low-glycemic and low-insulinemic indices, and its tooth-protective properties (for reviews, see [19,32]). Moreover, Shafer et al. [26] investigated the satiating potential of xylitol by measuring gastric emptying rates and energy intake during an *ad libitum* buffet. They were able to show that 25 g of xylitol significantly reduced energy intake compared to water; however, they did not compare it to other sweeteners. In our study, we compared various sweeteners and found no significant differences in energy intake between the four preloads, although the energy intake tended to be lower in response to xylitol compared to ace-K. Furthermore, both xylitol and ace-K resulted in lower total energy intake compared to sucrose. Another study examining the effect of a yoghurt sweetened with 25 g of xylitol on acute energy intake, both before and after a daily yoghurt consumption, found no significant differences in energy intake, but a reduction in total energy intake, compared to the sucrose-sweetened control yoghurt [27]. Teysseire et al. [25] investigated the effects of erythritol, another sugar alcohol with similar properties than xylitol, on subsequent *ad libitum* energy intake. They could show that erythritol lead to a lower energy intake compared to sucrose, sucralose, and water. Furthermore, total energy intake was lower in response to erythritol compared to sucrose, sucralose and water, which is comparable to our finding of a lower total energy intake in response to xylitol compared to sucrose. Consequently, despite variations in study designs, it has been demonstrated that xylitol exhibits similar satiating effects on energy intake compared to sucrose, while resulting in a lower total energy intake. This lower total energy intake may be attributed to the lower caloric content of xylitol compared to sucrose. While one might expect that the lower caloric content of xylitol would lead to a calorie compensation during the *ad libitum* test meal, this effect was not observed in our study. This absence of calorie compensation may be explained by the satiating properties of xylitol. The difference between the effects of erythritol and xylitol on energy intake indicates that erythritol might cause a stronger satiation effect, eventually mediated via the more pronounced release of GI satiation hormones in response to the erythritol preload [25].

Sucrose, as the most popular sugar, has been extensively studied for its satiating effects on energy intake and total energy intake. However, evidence regarding its satiating potential remains inconclusive. In an acute study by Farhat et al. [7], energy intake in response to a sucrose preload (60 g sucrose dissolved in 300 mL water) was similar compared to water, which is in line with the findings observed in the present study. However, total energy intake was not reported. Rolls et al. [8] observed a higher total energy intake after the inclusion of a sucrose drink in a meal compared to an aspartame drink or water. Other studies, depending on their study designs, reported either similar or a lower total energy intake in response to sucrose compared to artificial LC-HIS or water [5,9,10,11]. In the present study, total energy intake was similar in response to sucrose compared to water but higher compared to xylitol and ace-K.

Artificial low-caloric high-intensity sweeteners (LC-HIS) have been known for many years, with various studies investigating their effects on metabolism, including energy intake. Ace-K, in particular, has most often been studied in combination with other sweeteners, such as aspartame, rather than being administered alone. Two meta-analyses by Lee et al. [18] and Mehat et al. [17] summarized the effects of oral preloads sweetened with various artificial LC-HIS, including ace-K/aspartame combinations, on energy intake as well as total energy intake during subsequent *ad libitum* test meals. Lee et al. [18] reported that ace-K/aspartame-sweetened preloads led to higher subsequent energy intake compared to sucrose but similar energy intake compared to water. Moreover, both meta-analyses found that total energy intake in response to ace-K/aspartame was lower compared to sucrose, as only the sucrose-sweetened preloads contained calories, a finding consistent with our study [17,18]. Another study by Almiron-Roig et al. [33] showed that *ad libitum* (total) energy intake over a 24 h period was similar in response to preloads containing a sucralose/ace-K blend compared to sucrose.

The inconsistencies in findings regarding the effects of xylitol, sucrose, and ace-K can likely be attributed to variations in study designs, including differences in the administration of the sweetener and the timing of the test meals. Nevertheless, our findings indicate that oral preloads sweetened with xylitol or artificial LC-HIS result in lower total energy intake during subsequent *ad libitum* test meals, primarily due to their lower caloric content compared to sucrose.

The effects of xylitol on the release of GI satiation hormones, especially CCK, were confirmed in this study (for a review, see [19]). Notably, we observed a peak in CCK concentrations just before the *ad libitum* test meal, which may partially explain the absence of calorie compensation. Sucrose, as a carbohydrate, induces the release of the GI satiation hormones GLP-1 and GIP but has only a minor effect on plasma CCK concentrations [19]. We were able to show that CCK concentrations increased significantly in response to xylitol compared to sucrose. The LC-HIS ace-K does not affect the release of GI satiation hormones or gastric emptying [16,34].

It is noteworthy that xylitol is incompletely absorbed in the small intestine, with estimated absorption rates ranging from 50 to 95% [35]. Therefore, the exact amount of xylitol absorbed—and thus the number of calories it provides—remains uncertain. In this study, we calculated total energy intake based on a caloric content of 2.4 kcal/g for xylitol; however, the actual caloric intake may be lower, potentially leading to an even lower total energy intake compared to sucrose. The unabsorbed xylitol reaches the large intestine, where it can cause side effects such as bloating, borborygmi, and osmotic diarrhea, depending on the dose and individual sensitivity [36,37]. These side effects typically resolve within a few hours after consumption, which was also the case in this study. Notably, several studies have reported that chronic consumption of xylitol may lead to improved tolerance over time [37,38]. Moreover, some human intervention trials and *ex vivo* studies using human fecal matter found that xylitol may positively influence gut microbiota composition and leads to an increase in butyrate and propionate production [39,40,41,42,43,44,45]. Short-chain fatty acids like butyrate and propionate are beneficial in that they provide energy to the colonic epithelial cells, and play an important role in maintaining gut immunity, and support gut barrier function [46].

In this study, we also investigated the effects of the four preloads on parameters of glycemic control. The preload containing xylitol, similar to ace-K and water, did not affect plasma glucose concentrations before the *ad libitum* test meal but led to a small increase in plasma insulin concentration. These findings support previous studies demonstrating low or no acute effects of xylitol and ace-K on plasma glucose and insulin, respectively [14,19,47,48]. While most of these studies compared the effect of acute consumption of xylitol versus glucose, we showed that xylitol also has a significantly lower effect on plasma glucose and insulin compared to sucrose. Chronic consumption of xylitol does not appear to affect fasting glucose or insulin concentrations, while a chronic consumption of sucrose-sweetened beverages seems to reduce insulin sensitivity [19,49,50]. Chronic interventional studies examining the effects of ace-K on glycemic control are limited. Following a 12-week intervention with aspartame/ace-K-sweetened beverages, Bonnet et al. [51] found no significant effects on insulin sensitivity in healthy non-diabetic individuals. However, more chronic interventional studies are required to investigate the potential increased risk of developing type 2 diabetes mellitus (T2DM), as indicated by observational studies [21,22,52].

This randomized, controlled, double-blinded, crossover study provides insights into the effects of xylitol consumption on energy intake, GI satiation hormones, and glycemic control. The findings of this study have several potential implications for healthcare policy and clinical practice, particularly in the context of addressing obesity, diabetes, and metabolic syndrome. Given the global burden of these conditions, substituting high-caloric sugars like sucrose with low-caloric bulk sweeteners such as xylitol may represent a viable strategy to reduce overall energy intake without compromising taste or consumer acceptability. Our results show that xylitol consumption prior to meals leads to a lower energy intake and modulates postprandial insulin and glucose concentrations, highlighting its potential to support glycemic control and appetite regulation in individuals at risk for or living with type 2 diabetes. Some limitations should be acknowledged. In this trial, we only examined acute effects on energy intake. While we did not observe calorie compensation 15 min after preload consumption, we cannot exclude the possibility of compensation occurring within 24 h. Additionally, the effect of regular daily xylitol consumption on energy intake in a more realistic, everyday context requires further investigation.

## 5. Conclusions

In conclusion, our findings demonstrate that oral preloads sweetened with xylitol or the artificial LC-HIS ace-K followed by an *ad libitum* test meal result in lower total energy intake compared to a preload with sucrose, primarily due to their lower caloric content, but potentially also in part due to the CCK release found in response to xylitol.

## Figures and Tables

**Table 1:** The effects of the preloads containing xylitol, sucrose, ace-K, or water on energy intake (*ad libitum* test meal) and total energy intake (preload + *ad libitum* test meal) in 20 healthy participants ^1^.

Parameter	Main Effect of Preload	Planned Contrast Analyses					
		Xylitol vs. Sucrose	Xylitol vs. Ace-K	Xylitol vs. Water	Sucrose vs. Ace-K	Sucrose vs. Water	Ace-K vs. Water
Energy intake (kcal)	0.018 *	0.522	0.076 ^	0.171	0.899	0.847	0.989
Total energy intake ^2^ (kcal)	0.002 **	0.029 *	0.930	1.000	0.003 **	0.132	0.989

^1^ Statistics: linear mixed models followed by planned contrast analyses using post hoc Student’s *t*-tests with Tukey correction for multiple testing. ^2^ Total energy intake comprises calories from both the preload (xylitol preload: 80 kcal; sucrose preload: 134 kcal) and the *ad libitum* test meal. Ace-K, acesulfame potassium. Significance: ^ *p* < 0.1; * *p* < 0.05; ** *p* < 0.01.

**Table 2:** The effects of the preload containing xylitol compared to sucrose, ace-K, or water on GI satiation hormones, glycemic control, and appetite-related sensations in 20 healthy participants ^1^.

Parameter	Time Points	Main Effect of Preload	Preload-by-Time Interaction Effect	Planned Contrast Analyses		
				Xylitol vs. Sucrose	Xylitol vs. Ace-K	Xylitol vs. Water
CCK (pmol/L)	−1 min	0.012 *	<0.001 ***	<0.001 ***	<0.001 ***	<0.001 ***
	15 min			<0.001 ***	<0.001 ***	<0.001 ***
GLP-1_total_ (pmol/L)	−1 min	0.123	0.825	0.377	0.654	0.654
	15 min			0.693	0.473	0.473
Glucose (mmol/L)	−1 min	0.007 **	<0.001 ***	<0.001 ***	0.367	0.367
	15 min			<0.001 ***	0.510	0.510
Insulin (mIU/L)	−1 min	0.002 **	<0.001 ***	<0.001 ***	<0.001 ***	<0.001 ***
	15 min			0.172	0.032 *	0.059 ^
Hunger (cm)	−1 min	0.317	0.744	1.000	1.000	1.000
	15 min			0.909	0.909	0.909
Pfc (cm)	−1 min	0.179	0.666	1.000	1.000	1.000
	15 min			0.204	0.119	0.119
Satiety (cm)	−1 min	0.581	0.659	1.000	1.000	1.000
	15 min			1.000	1.000	1.000
Fullness (cm)	−1 min	0.195	0.850	0.620	0.516	0.620
	15 min			0.826	0.348	0.826

^1^ Statistics: linear mixed models followed by planned contrast analyses with Holm correction for multiple testing. Ace-K, acesulfame potassium; CCK, cholecystokinin; GLP-1, glucagon-like peptide-1; pfc, prospective food consumption. Significance: ^ *p* < 0.1; * *p* < 0.05; ** *p* < 0.01; *** *p* < 0.001.

**Table 3:** Assessment of GI tolerance in response to oral preloads containing xylitol, sucrose, ace-K, or water in 20 healthy participants.

Symptom	Number of Participants with Symptoms ^1^	Reported Severity ^2^
**Abdominal pain**		
Xylitol	1	1.0
Sucrose	0	0
Ace-K	0	0
Water	0	0
**Nausea**		
Xylitol	1	1.0
Sucrose	2	1.0
Ace-K	0	0
Water	0	0
**Vomiting**		
Xylitol	0	0
Sucrose	0	0
Ace-K	0	0
Water	0	0
**Diarrhea**		
Xylitol	3	1.0
Sucrose	0	0
Ace-K	0	0
Water	0	0
**Borborygmus**		
Xylitol	7	1.3
Sucrose	0	0
Ace-K	2	1.0
Water	2	1.0
**Bloating**		
Xylitol	2	2.0
Sucrose	0	0
Ace-K	0	0
Water	0	0
**Eructation**		
Xylitol	1	3.0
Sucrose	2	1.0
Ace-K	0	0
Water	1	1.0
**Flatulence**		
Xylitol	1	2.0
Sucrose	1	1.0
Ace-K	1	1.0
Water	1	1.0

^1^ GI symptoms were assessed by the use of a symptom check list. Participants were asked to rate each symptom as either “0, no symptom”, “1, mild symptom”, or “2, severe symptom”. ^2^ Reported severity was calculated by the sum of points divided by the participants with the symptom. Ace-K, acesulfame potassium; GI, gastrointestinal.

## Data Availability

The data described in this manuscript and codebook will be made publicly and freely available without restriction at https://github.com/labgas/proj_pfi-x (accessed on 28 January 2025).
